# Inflammasome Activation in Myeloid Malignancies—Friend or Foe?

**DOI:** 10.3389/fcell.2021.825611

**Published:** 2022-01-27

**Authors:** Nicola Andina, Nicolas Bonadies, Ramanjaneyulu Allam

**Affiliations:** ^1^ Department of Hematology and Central Hematology Laboratory, Inselspital Bern University Hospital, University of Bern, Bern, Switzerland; ^2^ Department for BioMedical Research, University of Bern, Bern, Switzerland

**Keywords:** NLRP3 inflammasome, myeloid malignancies, myelodysplastic syndromes, myeloproliferative neoplasms, chronic myeloid leukemia, acute myeloid leukemia and targeting inflammasomes

## Abstract

Myeloid malignancies including myelodysplastic syndromes, myeloproliferative neoplasms and acute myeloid leukemia are heterogeneous disorders originating from mutated hematopoietic stem and progenitor cells (HSPCs). Genetically, they are very heterogeneous and characterized by uncontrolled proliferation and/or blockage of differentiation of abnormal HSPCs. Recent studies suggest the involvement of inflammasome activation in disease initiation and clonal progression. Inflammasomes are cytosolic innate immune sensors that, upon activation, induce caspase-1 mediated processing of interleukin (IL) -1-cytokine members IL-1β and IL-18, as well as initiation of gasdermin D-dependent pyroptosis. Inflammasome activation leads to a pro-inflammatory microenvironment in the bone marrow, which drives proliferation and may induce clonal selection of mutated HSPCs. However, there are also contradictory data showing that inflammasome activation actually counteracts leukemogenesis. Overall, the beneficial or detrimental effect of inflammasome activation seems to be highly dependent on mutational, environmental, and immunological contexts and an improved understanding is fundamental to advance specific therapeutic targeting strategies. This review summarizes current knowledge about this dichotomous effect of inflammasome activation in myeloid malignancies and provides further perspectives on therapeutic targeting.

## Introduction

Inflammation is the evolutionary conserved response to pathogens or injury. Although it is beneficial to fight against infection and wound healing, undesired chronic inflammation is linked to several noncommunicable disorders including diabetes, cardiovascular diseases, and cancer (Coussens and Werb, 2002; Furman et al., 2019). It is very well accepted that inflammation predisposes to growth of solid tumors, which represents one of the hallmarks of cancer (Colotta et al., 2009). More recently, the role of inflammation in hematological malignancies has gained momentum (Gañán-Gómez et al., 2015; Monlish et al., 2016; Craver et al., 2018; Sallman and List, 2019; Ratajczak et al., 2020). Myeloid malignancies, including myelodysplastic syndromes (MDS), myeloproliferative neoplasms (MPN) and acute myeloid leukemia (AML), are heterogeneous disorders originating from mutated hematopoietic stem and progenitor cells (HSPCs). Genetically, they are very heterogeneous and characterized by uncontrolled proliferation and/or blockage of differentiation in the bone-marrow (BM) of abnormal HSPCs (Corey et al., 2007; Döhner et al., 2015; Tefferi and Pardanani, 2015). With the expression of pattern recognition receptors (PRRs) and cytokine receptors, HSPCs can directly sense pathogen-associated molecular patterns (PAMPs) and damage-associated molecular patterns (DAMPs). These mechanisms are part of innate immunity, allow activation of pro-inflammatory cytokines to increase defense against emerging danger, and the signals eventually affect the fate choice of HSPCs as well as differentiation (King and Goodell, 2011). Recently, several studies suggested a causative role of the innate immune system mediated inflammation in the pathogenesis and progression of myeloid malignancies. Multiple key regulators of innate immunity such as toll-like receptors (TLRs) and their downstream signaling molecules, TNFR1, TNFR2 and CD95 are overexpressed or constitutively activated in HSPCs (Gañán-Gómez et al., 2015; Barreyro et al., 2018; Camacho et al., 2021). Dysregulation of these molecules leads to abnormal hematopoiesis, unbalanced cell death and proliferation in patients’ BM, implying an important role in the pathogenesis of myeloid malignancies. Inflammasomes are central mediators of inflammation and growing evidence suggest their involvement in cancer progression (Karki and Kanneganti, 2019). On the contrary, few studies also suggest a beneficial role for inflammasomes in cancer (Kolb et al., 2014; Hamarsheh and Zeiser, 2020). In this review, we summarize current knowledge about this dichotomous effect of inflammasome activation in myeloid malignancies and provide further perspectives on therapeutic targeting.

## Biology of Inflammasomes

### Pattern Recognition Receptors

The innate immune system engages an array of germline encoded PRRs to detect the presence of microorganisms or tissue damage. PRRs recognize structures conserved among microbial species or endogenous molecules released from tissue damage and cell death, which are called PAMPs and DAMPs, respectively. Currently, there are four different classes of PRR families that includes transmembrane proteins such as the Toll-like receptors (TLRs) and C-type lectin receptors (CLRs), as well as cytoplasmic proteins such as the Retinoic acid-inducible gene (RIG)-I-like receptors (RLRs) and Nucleotide-binding oligomerization domain (NOD)-like receptors (NLRs) (Takeuchi and Akira, 2010). Compared to all the other innate immune receptor molecules, the NLRs appear to be predominantly involved in chronic non-infectious sterile inflammation (Schroder and Tschopp, 2010). Among the NLR family, several NLRP (NOD, leucine rich repeat and pyrin domain containing) proteins form inflammasome complexes (Martinon et al., 2009).

### Different Types of Inflammasomes

**FIGURE 1:**
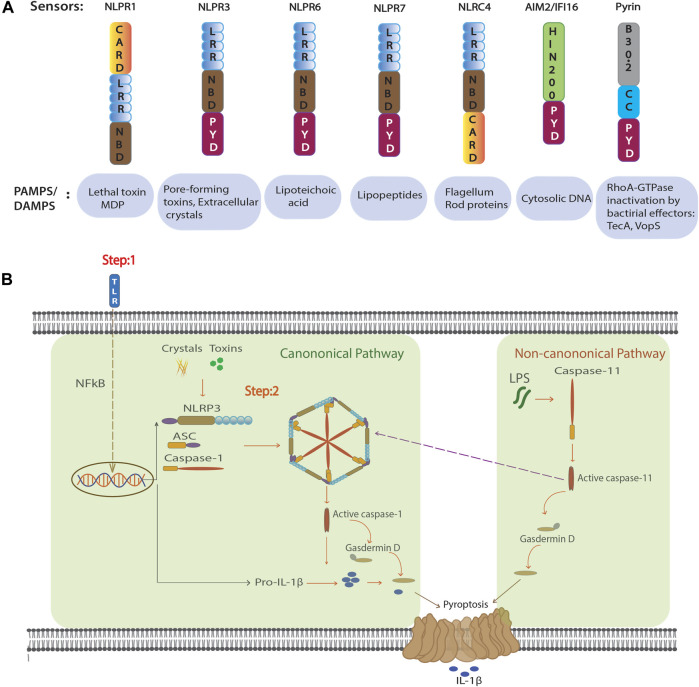
Inflammasome configuration and activation. **(A)** Domain structure of the NLR proteins that form active inflammasomes and non-NLR inflammasome AIM2 and Pyrin (above). Known activators of inflammasome formation (below). **(B)** Suggested activation scheme for canonical and non-canonical NLRP3 inflammasome formation. In the canonical pathway, NF-κB–activation by TLR ligation in the first step increases transcription and translation of NLRP3 and pro-IL-1β. In the second step, NLRP3 agonists such as MSU crystals or pore forming toxins induce inflammasome formation and thus allow autocleavage and activation of caspase-1. Matured caspase-1 induces cellular activation by cleavage of the cytokines IL-1β and IL-18, and GSDMD mediated pyroptosis. In non-canonical pathway intracellular LPS binds and activates caspase-11. Active form of caspase-11 mediates GSDMD-dependent pyroptosis and further activation of NLRP3 inflammasome.

Inflammasomes are multiprotein complexes that activate inflammatory caspases such as Caspase-1. They process the precursor molecules of IL-1 cytokine members IL-1β and IL-18 and induce a Gasdermin D (GSDMD)-dependent lytic form of cell death called pyroptosis (Broz and Dixit, 2016). Several NOD-like receptors (NLRs), as well as HIN200 proteins, can form inflammasome complexes, namely: NLRP3, NLRP1, NLRP6, NLRC4, NAIP, AIM2, and Pyrin (Schroder and Tschopp, 2010; Broz and Dixit, 2016; Heilig and Broz, 2018). They are components of the innate immune system and are activated by different PAMPs and DAMPs (Broz and Dixit, 2016) (Figure 1A). Recognition of an inflammatory ligand leads to oligomerization of the sensor protein and recruitment of the adaptor protein ASC (apoptosis associated speck-like protein). ASC contains two death fold domains: one pyrin domain (PYD) and one caspase recruitment domain (CARD) (Martinon et al., 2009). ASC interacts with the upstream inflammasome sensor protein *via* its pyrin (PYD) domain. Moreover, with the help of its CARD domain it brings monomers of pro-caspase-1 into close proximity, which initiates caspase-1 self-cleavage and the formation of active caspase-1 (Broz and Dixit, 2016). Active caspase-1 proteolytically activates pro-IL-1β and pro-IL-18 and induces GSDMD mediated pyroptosis **(** Figure 1B). The best understood inflammasome is NLRP3, which recognizes numerous exogenous and host ligands (Swanson et al., 2019).

### NLRP3 Inflammasome Activation

Canonical NLRP3 inflammasome activation is a two-step mechanism (Latz et al., 2013). The first step is a priming step and involves a NF-κB–activating stimulus, such as lipopolysaccharide (LPS) binding to the toll-like receptor (TLR)-4 that induces transcription and translation of pro-IL-1β, as well as increased expression of NLRP3. In a second step, various triggers such as nigericin, monosodium urate crystals (MSU) (and others) activate NLRP3 to assemble the inflammasome and activate caspase-1 (Figure 1B). Several mechanisms have been reported to activate NLRP3, including potassium (K+) efflux (Pétrilli et al., 2007), lysosomal destabilization (Hornung et al., 2008), and mitochondrial ROS production (Zhou et al., 2010). Recently NEK7, a member of the family of mammalian NIMA-related kinases (NEK proteins) was shown to be an essential mediator of NLRP3 activation (He et al., 2016). However, to date, no master regulator has been found to connect various activators to NLRP3 inflammasome activation. In addition, NLRP3 can be activated through a non-canonical pathway that involves caspase-11. Intracellular LPS from Gram-negative bacteria can directly bind and activate caspase-11 (Figure 1B). This activation leads to pyroptosis through cleavage of GSDMD and triggers a secondary activation of the canonical NLRP3 inflammasome (Kayagaki et al., 2011; Shi et al., 2014). Recently, it has been shown that LPS alone can directly activate NLRP3 inflammasome independent of a second signal in human monocytes known as alternative inflammasome activation. This pathway is dependent on caspase-8 downstream of TLR4 and does not activate pyroptosis (Gaidt et al., 2016; Gaidt and Hornung, 2017).

## Role of Inflammasomes in Myeloid Malignancies

Hematopoiesis is a process that leads to the production of billions of mature and terminally differentiated peripheral blood cells every day from a few HSPCs that reside in the BM. It is a complex and tightly regulated process involving hematopoietic cytokine receptor signaling (Baker et al., 2007). Myeloid malignancies are clonal disorders that arise from mutated HSCs and comprised chronic disorders, such as MDS, MPN, myelodysplastic/myeloproliferative “overlap” neoplasms (MDS/MPN), which all have the potential to transform to overt AML. Myeloid malignancies are genetically and phenotypically heterogeneous disease entities, and the prognosis is mainly determined by the inherent risk of transformation to AML (Beham-Schmid and Schmitt-Graeff, 2020). Overall, men are slightly more affected than women and the incidence rate increases with age (Hehlmann et al., 2007; Rohrbacher and Hasford, 2009; Neukirchen et al., 2011; Juliusson et al., 2012; Moulard et al., 2014; Bonadies et al., 2017; Benzarti et al., 2019). MPN are characterized by increased production of myeloid cells, while MDS are characterized by ineffective hematopoiesis with dysplasia and cytopenia (Beham-Schmid and Schmitt-Graeff, 2020). MDS/MPN neoplasms share features of both MDS and MPN, where chronic myelomonocytic leukemia (CMML) is the most frequent entity of these overlap syndromes. CMML is characterized by myelomonocytic proliferation with persistent monocytosis, myeloid dysplasia and ineffective hematopoiesis leading to cytopenias (Beham-Schmid and Schmitt-Graeff, 2020). AML is a biologically heterogeneous disease characterized by a more aggressive course of the disease with increased production of mainly very immature cells called “blasts” (Beham-Schmid and Schmitt-Graeff, 2020). In all these myeloid malignancies, the myeloid compartment, in which the inflammasomes play an essential role, is severely affected, with an important impact on the regulation of inflammasome activation.

### Involvement of Inflammasome Mediated Pyroptosis in MDS

Several clinical and molecular studies suggested that abnormal activation of innate immune signals and associated inflammation contribute to the pathogenesis of MDS. Abnormal levels of cytokines (e.g., TNF, IL-6, IL-8), chemokines (e.g. CXCL5, CCL3, CCL2) and growth factors (e.g., GM-CSF, VEGF, M-CSF) in the peripheral blood and bone marrow of patients with MDS have been observed, and increased levels of these cytokines can affect the clinical outcomes of patients (Gañán-Gómez et al., 2015). Furthermore, larger-scale epidemiological studies have demonstrated that patients with autoimmune disorders have an increased risk of developing MDS (Dalamaga et al., 2002; Anderson et al., 2009). Moreover, about 20–30% of MDS and MDS/MPN patients present with a variety of systemic autoimmune and autoinflammatory manifestations and it remains to be elucidated, if they are causative involved in the pathogenesis or just “innocent” consequences because of a deregulated immune system (Kipfer et al., 2018). Increased intramedullary cell death has been reported to be one of the main causes of the cytopenias that characterize MDS (Gañán-Gómez et al., 2015). However, the key inflammatory mediators responsible for innate immune activation and cell death remain unknown. Interestingly, few studies have reported that caspase-1 is activated in bone marrow cells from MDS patients (Mundle et al., 1996; Boudard et al., 2000), suggesting an involvement of inflammasome activation. In line with this, Basiorka et al. reported involvement of NLRP3 inflammasome in MDS (Basiorka et al., 2016). The DAMP proteins S100A8 and S100A9 are the key mediators for activation of NLRP3 inflammasome (Simard et al., 2013). The same group showed that S100A9 is particularly overexpressed in MDS HSPCs, and it induces increased ROS production. This ultimately leads to activation of NLRP3 inflammasome, cation influx, cell swelling, and beta-catenin activation. Inhibition of NLRP3 with the icariin derivative ICTA rescued the MDS phenotype in S100A9 transgenic (S100A9^Tg^) mice (Basiorka et al., 2016). Although this study establishes the NLRP3 inflammasome as a central player in the pathology of MDS, further research is needed to confirm the precise *in vivo* role of the NLRP3 inflammasome in the development and progression of MDS. For example, ICTA is not a specific NLRP3 inflammasome inhibitor and has anti-inflammatory and antitumor effects and also modulates myeloid-derived suppressor cells (MDSCs) (Zhou et al., 2011). Furthermore, only 20% of S100A9^Tg^ transgenic mice showed an MDS-like phenotype after 9–12 months of age, suggesting additional contributing cofactors that play a role in MDS development in this mouse model (Chen et al., 2013) (personal communication with the Sheng Wei group and our own observations). Additional studies with NLRP3 specific inhibitors or using NLRP3 knockout mice in MDS mouse models such as SRSF2 (Kim et al., 2015) and SFB1 (Obeng et al., 2016) will clarify the role of NLRP3 in MDS.

It is apparent that pyroptosis plays a critical role in MDS pathology (Sallman and List, 2019). In addition to NLRP3 inflammasome, other inflammasomes and inflammasome-independent pathways can also induce pyroptosis (Man et al., 2017; Yu et al., 2021) and may be involved in MDS. For example, NLRP1a inflammasome is highly expressed in HSPCs and over activation leads to pyroptosis of HSPCs resulting in leukopenia, bone marrow hypoplasia, and immunosuppression (Masters et al., 2012). Furthermore, the death receptor Fas and its specific ligand (Fas-L), is overexpressed and correlates with the rate of apoptosis in MDS (Bouscary et al., 1997; Gersuk et al., 1998; Gupta et al., 1999), induces inflammasome independent non-canonical IL-1β and IL-18 maturation and cell death *via* caspase-8 (Bossaller et al., 2012). Recently, caspase-8 has been shown to cleave GSDMD and activate pyroptosis (Sarhan et al., 2018). Collectively, these studies suggest that in addition to the NLRP3 inflammasome, other inflammasomes as well as inflammasome independent pathways could play a role in the pathology of MDS. Further detailed molecular and genetic studies are necessary to understand the role of inflammasome activation in MDS and to identify which inflammasome signaling plays a role upstream of caspase-1 activation and at which state of the disease.

### Inflammasomes in MPN

Classical MPNs comprise polycythemia vera (PV), essential thrombocythemia (ET), primary myelofibrosis (PMF), and prefibrotic PMF (Swerdlow et al., 2017). The most frequent molecular alteration that can be found in these entities is a point mutation in exon 14 of the janus kinase 2 (*JAK2*) gene (*JAK2* ^*V617F*^) (Baxter et al., 2005; James et al., 2005; Kralovics et al., 2005; Levine et al., 2005). While PV is almost exclusively based on a *JAK2* driver mutation, ET and PMF also show mutations of calreticulin (*CALR*) and of the thrombopoietin receptor gene (*MPL*), with some triple-negative cases (Swerdlow et al., 2017). Cell intrinsic genetic alterations in HSPCs play a pivotal role in the pathogenesis of myeloid malignancies. However, the interaction between the malignant cell and niche of the HSPCs is also crucial (Korn and Méndez-Ferrer, 2017). Niches are local microenvironments that maintain and regulate stem cell biology (Morrison and Scadden, 2014). An intact, physiologic stem cell niche can inhibit proliferation of malignant HSPC (Arranz et al., 2014). On the other hand, a malignant cell can remodel the niche for its own benefit and for the disadvantage of normal HSPCs (Asada et al., 2017). Interestingly, the pro-inflammatory cytokine IL-1β is involved in the remodeling of the niche and is also reported to be involved in the pathogenesis of several classical MPNs, such as polycythemia vera, essential thrombocythemia, and primary myelofibrosis (Arranz et al., 2014; Asada et al., 2017; de Mooij et al., 2017). In line with this, a recent study showed that IL-1β supports clonal expansion of Jak2 mutated HSPCs in MPN mice and deletion of IL-1β in these mice reduces disease burden (Rai et al., 2019). Interestingly, increased expression of the AIM2 inflammasome has been reported in Jak2 mutant cells (Liew et al., 2016). Furthermore, Fidler et al. showed that *Jak2* ^*V617F*^ macrophages express an increase in AIM2 inflammasome activation and deletion of the AIM2 but not NLRP3 gene rescued the atherosclerosis phenotype driven by *Jak2* ^*V617F*^ clonal hematopoiesis (Fidler et al., 2021). However, it needs to be further evaluated whether AIM2 is involved in the *Jak2* ^*V617F*^ driven MPN phenotype.

### Inflammasomes in CML

In chronic myeloid leukemia (CML), the genetic basis is a translocation that juxtaposes the abelson tyrsoin kinase gene (*ABL1*) from chromosome 9 to the breakpoint cluster region (*BCR*) on chromosome 22. The resulting fusion gene *BCR-ABL1* gives rise to the BCR-ABL fusion protein and is disease defining for CML with proliferative and survival advantage of the cell (Quintás-Cardama and Cortes, 2009). Before the development of highly specific tyrosine kinase inhibitors (TKI) against BCR-ABL, CML was a life-threatening disease and can today be controlled with a life expectancy close to normal. However, leukemic stem cells (LSC) in CML are not dependent on BCR-ABL activity and persist despite TKI therapy. Therefore, more relevant targets for these LSCs are being investigated. Interestingly, CML leukemic stem cells (LSC) show increased expression of IL-1R1 and its co-receptor IL1RAP (Zhang et al., 2016; Ågerstam et al., 2016). Activation of the IL-1β/IL-1R1 signaling pathway increases proliferation of CML LSC and antibodies against IL1RAP suppress IL-1β induced proliferation. Furthermore, in CML mouse models, inhibition of the IL-1β/IL-1R1 signaling pathway increases the effect of TKIs.

### Inflammasome Activation in MDS/MPN Overlap Syndromes

A recent study by Hamarsheh et al. showed involvement of NLRP3 inflammasome in *Kras* ^*G12D*^ mutation driven myeloid leukemia (Hamarsheh et al., 2020). They demonstrate that the *Kras* ^*G12D*^ mutation in HSPCs developed a myeloproliferative and myelodysplastic phenotype, reminiscent of CMML, and mice lacking the *Nlrp3* gene in *Kras* ^*G12D*^ mice reversed this phenotype. Additionally, treatment with either IL-1 receptor blockade or NLRP3 inhibitor (MCC950) reduced myeloproliferation. Supporting this, they found increased caspase-1 activation and IL-1β production in CMML, juvenile myelomonocytic leukemia (JMML) and AML patients with KRAS mutations as compared to patients without KRAS mutations (Hamarsheh et al., 2020). However, non-KRAS mutant cells showed caspase-1 activation and IL-1β production, albeit at lower levels as compared to KRAS mutant cells (Hamarsheh et al., 2020). Further studies are needed to evaluate which inflammasome is responsible for caspase-1 activation in non-KRAS mutant leukaemia patients.

### Role of Inflammasome Mediated IL-1β in AML

Similarly to MPN, IL-1β promotes the growth of malignant HSPCs in AML and inhibits normal HSPCs by altering the bone marrow niche (Carey et al., 2017; Wang et al., 2020). Furthermore, AML LSCs also showed increased expression of IL-1R1 and its co-receptor IL1RAP (Askmyr et al., 2013). Activation of the IL-1β/IL-1R1 signaling pathway increased AML LSC proliferation and antibodies against IL1RAP suppressed IL-1β induced proliferation (Askmyr et al., 2013; Ågerstam et al., 2015). There is, however, also evidence that inflammasome activation counteracts leukemogenesis. The presence of FMS-like tyrosine kinase 3 (FLT3) mutations and especially FLT3-internal tandem duplications (FLT3-ITD) are associated with poor prognosis in AML and contribute to proliferation of the malignant clone (Chu et al., 2012; Mead et al., 2013). In a FLT3-ITD AML mouse model, receptor-interacting protein kinase 3 (RIP3K) restricts myeloproliferation *via* inflammasome mediated IL-1β secretion (Höckendorf et al., 2016). Similar findings for RIP3K were observed with the oncogenic fusion protein AML1-ETO but not for MLL-ENL-induced leukemia (Höckendorf et al., 2016), suggesting that RIP3K- mediated inflammasome activation and IL-1β secretion play a counteracting role in some but not all AML subtypes. In addition, many drugs that are used for cancer treatment also induce IL-1β production through inflammasome activation (Bent et al., 2018). For example, cytarabine used to treat AML (Löwenberg et al., 2011) induces IL-1β secretion through NLRP3 (Wong et al., 2014). Collectively, these data suggest that the effect of inflammasome mediated IL-1β secretion on leukemogenesis depends on the mutational context and can either favor or counteract leukemia.

### Role of Inflammasome Independent IL-1β Secretion in Myeloid Malignancies

As mentioned above, IL-1β plays a crucial role in a variety of myeloid malignancies (Arranz et al., 2017). Although, mainly inflammatory caspases from inflammasomes cleave pro-IL-1β into its active form IL-1β, there is evidence that pro-IL-1β can be processed by inflammasome-independent mechanisms (Netea et al., 2014). Proteases, such as proteinase 3 (PR3), elastase, cathepsin G, chymase, and chymotrypsin, are expressed by neutrophils and macrophages and cleave pro-IL-1β (Netea et al., 2014). In addition, also metalloproteinases, such as meprins cleave pro-IL-1β (Broder and Becker-Pauly, 2013). Interestingly, some of these proteins are highly expressed in myeloid malignancies. For example, cathepsin G is highly expressed in AML blasts and was proposed as a therapeutic target (Alatrash et al., 2017). Furthermore, neutrophil elastase is also increased in the serum of patients with AML (Sergeeva et al., 2006). Whether these proteases significantly contribute to pro-IL-1β processing in myeloid malignancies is currently unknown.

## Targeting Inflammasomes to Treat Myeloid Malignancies

As inflammasomes are involved in myeloid malignancies, they represent attractive candidates for drug targeting. Indeed, several NLRP3 inhibitors have been developed and some are in clinical trials to treat inflammatory disorders and cancer (Mangan et al., 2018; Sallman and List, 2019; Hamarsheh and Zeiser, 2020). For example, the bruton tyrosine kinease (BTK) inhibitor ibrutinib binds directly to ASC and NLRP3, and inhibits the activation of inflammasome (Weber, 2021). This compound is currently in phase I clinical trials for high-risk MDS (Sallman and List, 2019). Further, MCC950 is a specific NLRP3 inhibitor and represents a promising molecule to target NLRP3 mediated disease progression in myeloid malignancies (Coll et al., 2015). In addition to NLRP3 inhibitors, targeting IL-1β is more advanced in clinical trials. There are currently three proteins approved for inflammatory diseases targeting IL-1 signaling and are under investigation for MDS treatment (Sallman and List, 2019). These are i) rilonacept, a soluble decoy receptor that binds both IL-1β and IL-1α, ii) anakinra, an antagonist of the IL-1 receptor, and iii) canakinumab, an IL-1β neutralizing antibody (Mangan et al., 2018). However, targeting IL-1 signaling alone will not inhibit other pro-inflammatory consequences of inflammasome activation, such as IL-18 and HMGB1 production and pyroptosis. Especially in MDS, where pyroptosis might be responsible for ineffective hematopoiesis and cytopenia, inflammasome targeting up-stream of IL-1β, at the level of caspase-1 or higher, could have an additional beneficial effect compared to targeting IL-1β alone. However, in AML, inflammasome-mediated pyroptosis eliminates malignant clones (Höckendorf et al., 2016) and inhibition of inflammasome-mediated pyroptosis could, therefore, potentially also increase disease burden (Höckendorf et al., 2016). In summary, targeting components of the inflammasome to treat myeloid malignancies could be an interesting additive to existing therapies. However, due to its dichotomous role in myeloid malignancies, more knowledge about the mechanisms is indispensable to understand in which context inflammasome targeting might be beneficial or deleterious.

## Conclusion

There has been enormous progress for the past decade in understanding the molecular mechanisms of inflammasome activation and its role in myeloid malignancies. However, more efforts are needed to understand comprehensively the context-dependent and dichotomous role of inflammasomes in myeloid malignancies. Since myeloid malignancies are genetically heterogeneous and various genetic mutations seem to converge in inflammasome activation, modulating inflammasome activity could provide a targeted approach to a specific pathway with a larger impact on HSCs with different mutations.
